# Treatment of abdominal aortic pseudoaneurysm caused by brucellosis with endovascular aneurysm repair

**DOI:** 10.3389/fbioe.2023.1122997

**Published:** 2023-01-19

**Authors:** Xiao Li, Qilong Wang, Yang Zhang, Xiwei Sun, Hang Yin, Hua Zhang, Sean X. Luo, Zhongying Wang, Qi Yu, Zhiming Chen, Zhihua Cheng

**Affiliations:** ^1^ Department of Vascular Surgery, General Surgery Center, The First Hospital of Jilin University, Changchun, China; ^2^ Department of Forensic Medicine, Basic Medical College, Jilin University, Changchun, China

**Keywords:** brucellosis, abdominal aortic pseudoaneurysm, peripheral arterial disease, digital subtraction angiography, endovascular aneurysm repair

## Abstract

Peripheral vascular disease caused by brucellosis is rarely seen around the world; thus, it is easily ignored by patients and doctors, leading to a lack of corresponding screening and delayed comprehensive treatment. Currently, there is no standard or guideline for diagnosing and treating peripheral arterial disease caused by brucellosis. From June 2021 to December 2022, four cases of abdominal aortic pseudoaneurysm caused by brucellosis disease were treated with endovascular aneurysm repair This study reported treatment results as follows and reviewed the incidence, treatment, and prognosis of abdominal aortic pseudoaneurysm caused by brucellosis.

## Introduction

Brucellosis is one of the world’s most common zoonotic infectious diseases (Ariza et al., 2007). It is mainly transmitted to humans by ingesting infected dairy products or meat or through direct contact with infected animals (Carabin et al., 2012). Therefore, this disease primarily occurs in Middle Eastern, Mediterranean, and East Asian countries, and the bacterium reappears in developed countries through international travel, migration, and international food trade. The incidence of brucellosis is increasing yearly due to the successful development of animal husbandry in China, which increases the chance of human infection (Jiang et al., 2020). Brucellosis has a wide range of clinical manifestations, ranging from acute indistinguishable febrile illness to chronic infectious disease that most often affects the central nervous, cardiovascular, or skeletal systems (Willems et al., 2022). Although cardiovascular involvement in brucellosis is rare, complications due to cardiovascular involvement are a common cause of death from brucellosis. Peripheral artery pseudoaneurysms caused by brucellosis are very rare. We have previously reported a case of a thoracic aortic pseudoaneurysm (Wang et al., 2017) and a case of an iliac aneurysm (Wang et al., 2021). In the case of a thoracic aortic pseudoaneurysm with brucellosis involvement, we used EVAR to exclude the thoracic aortic pseudoaneurysm. Post-operative patients were treated with 450 mg rifampin twice a day orally and 200 mg doxycycline once a day orally for 6 months to combat brucellosis. We had a 6-month follow-up, and the patient was healthy and asymptomatic. A recently published review showed that in the peripheral vasculature, brucellosis most frequently affects the abdominal and thoracic aorta, with iliac and other peripheral arteries more rarely involved (Willems et al., 2022). Here, we report four cases of abdominal aortic pseudoaneurysm caused by brucellosis: one patient was previously diagnosed with brucellosis before admission, the other three patients were diagnosed at examination after abdominal aortic pseudoaneurysm was detected, and all four patients were treated with an EVAR technique.

## Clinical cases

**FIGURE 1:**
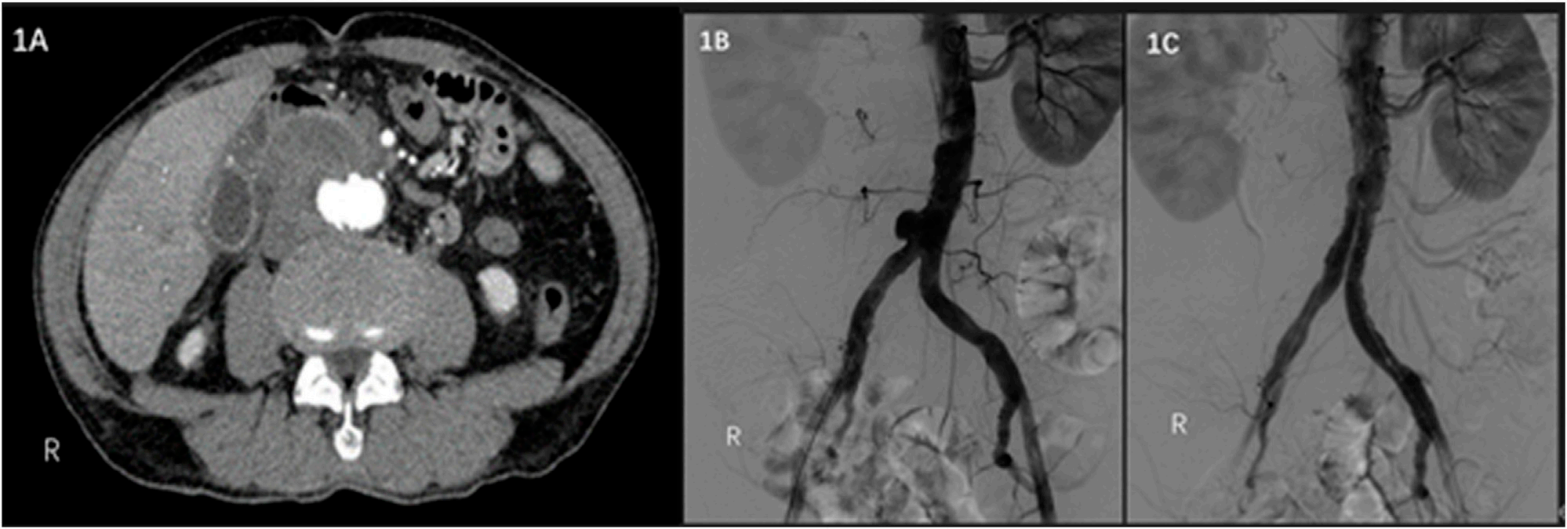
**(A)** Contrast-enhanced CT scan of the abdomen shows a pseudoaneurysm at the end of the abdominal aorta with local encapsulation. **(B)** Intraoperative DSA shows a pseudoaneurysm at the end of the abdominal aorta. **(C)** Intraoperative DSA shows no endoleaks.

A 79-year-old male was admitted to a hospital on 17 February 2021 due to sudden severe abdominal pain. Enhanced computed tomography (CT) examination of the abdomen showed that the distal end of the abdominal aorta showed significant ballooning, which was considered an aneurysm with mural thrombus or hematoma formation, and the rupture might increase (Figure 1A). The patient’s past medical history showed no history of hypertension, diabetes, coronary heart disease, surgery, trauma, drug abuse, hepatitis B, and other infectious diseases. Regarding previous work history, the patient worked and was exposed to cattle and sheep for more than 20 years. The tests showed C-reactive protein (CRP) of 125.48 mg/L, erythrocyte sedimentation rate (ESR) of 84 mm/h, white blood cell count (WBC) of 8.01 × 10^12^/L, neutrophil percentage of 68%, and hemoglobin (Hb) of 119 g/L. Considering the patient’s previous work history of contact with cattle and sheep, brucellosis was not excluded, and a bacterial blood culture was performed. On the same day, emergency endovascular repair of the abdominal aortic aneurysm was performed, and intraoperative digital subtraction angiography (DSA) revealed pseudoaneurysm formation at the distal end of the abdominal aorta (Figure 1B). Endovascular repair of the aneurysm was completed using ENDURANT-covered main body stents (23 mm–13 mm–145 mm; Medtronic, Minneapolis, MN, United States) and iliac branch stents (16 mm–13 mm–93 mm), with the proximal end starting at the inferior margin of the lowest renal artery and covering to the lots of the bilateral common iliac arteries. A 6 F sheath (Terumo Corporation, Japan) was retrogradely inserted into the left common femoral artery intraoperatively. On digital subtraction angiography with a pigtail catheter, a pseudoaneurysm was identified (Figure 1B). Then, with the help of a Lunderquist Extra-Stiff Wire Guide (Cook Medical Inc. Denmark), an ENDURANT-covered main body stent (23 mm–13 mm–145 mm; Medtronic, Minneapolis, MN, United States) and an iliac branch stent (16 mm–13 mm–93 mm) were introduced and implanted to isolate the pseudoaneurysm. On repeat angiography, the terminal abdominal aortic pseudoaneurysm was not visualized, and no endoleaks were seen (Figure 1C). *Brucella* was isolated from the patient’s blood after the operation, hence, the patient was diagnosed with brucellosis. The standard tube agglutination test (SAT) result was 1:50, which met the solid positive result. Rifampicin was given for anti-*Brucella* treatment after surgery. Ultrasound Doppler examination was performed 6 months after the surgery, the stent morphology was good, and no aneurysm change was found at both ends of the covered stent. During the follow-up visit 1 year after surgery, the patient’s family informed that the patient died of hemorrhagic shock due to sudden gastrointestinal bleeding of unknown cause 10 months after surgery.

**FIGURE 2:**
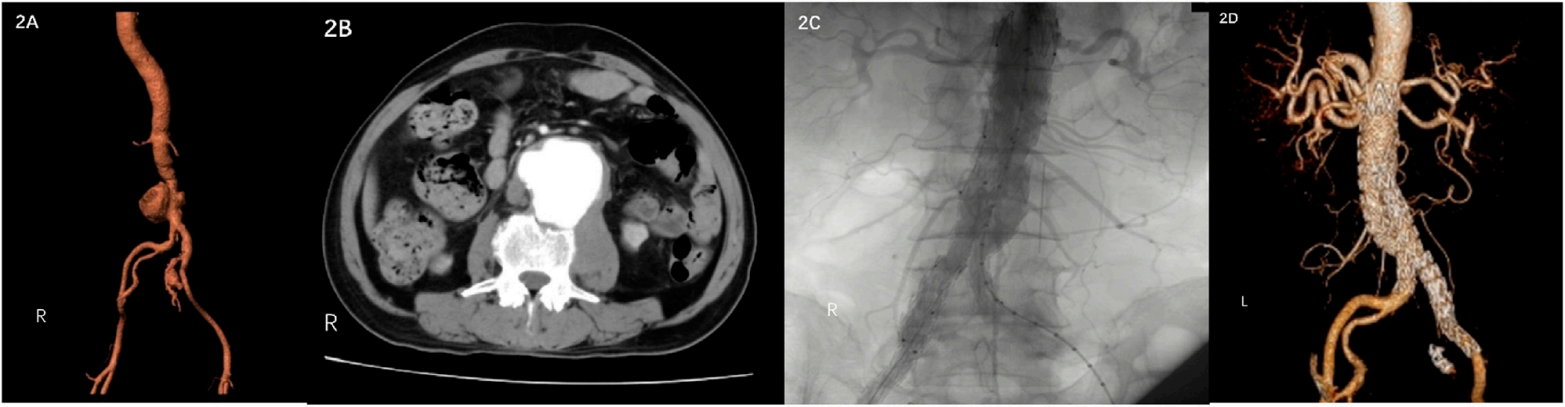
**(A) (B)** Contrast-enhanced CT showed pseudoaneurysm formation in the middle and lower abdominal aorta and right internal iliac artery. **(C)** DSA showed no endoleaks. **(D)** CTA was reexamined 5 months after the operation.

A 67-year-old male visited the hospital on 9 June 2022 due to low back pain for 1 month, which increased for 1 day. The patient had a history of gout for more than 10 years, without a history of drug abuse, abdominal surgery, trauma, hypertension, diabetes, and coronary heart disease. Contrast-enhanced abdomen CT revealed a pseudoaneurysm of the distal abdominal aorta with a maximum diameter of 65.4 cm and a pseudoaneurysm of the right internal iliac artery (Figure 2A 2 B). The tests demonstrated CRP of 36.75 mg/L, ESR of 107 mm/h, WBC of 5.04 × 10^12^/L, neutrophil percentage of 88%, and Hb of 110 g/L. The patient reported working on a farm, and a bacterial blood culture was performed. Due to significant abdominal pain, the patient underwent emergency endovascular exclusion of the abdominal aorta, and the right internal iliac artery was embolized. Intraoperative DSA showed the formation of abdominal aortic and right internal iliac artery pseudoaneurysm. The right internal iliac artery was embolized with three 5 mm–10 mm MReye Embolization Coils (Cook Medical, Bloomington, IN, United States) and five 5 mm–8 mm MReye Embolization Coils (Cook Medical, Bloomington, IN, United States). ENDURANT-covered stent (28 mm–16 mm–145 mm; Medtronic, Minneapolis, MN, United States) and iliac branch stent (16 mm–13 mm–124 mm) were used to complete endovascular exclusion of the aneurysm. The proximal end was protected from the lower edge of the most inferior renal artery to the ends of bilateral common iliac arteries. On repeat angiography, the terminal abdominal aortic pseudoaneurysm was not visualized, and no endoleaks were seen (Figure 2C). *Brucella* was isolated from blood bacterial culture after the operation, and brucellosis was diagnosed. The SAT result was 1:100, which met the solid positive result. Anti-brucellosis treatment was given to the patient. The patient’s abdominal pain disappeared 1 week after the surgery. The patient’s reexamination of the thoracoabdominal aorta by CTA 5 months after the surgery showed that the stent in the abdominal aorta and bilateral iliac arteries was in good shape without obvious abnormality and migration (Figure 2D).

**FIGURE 3:**
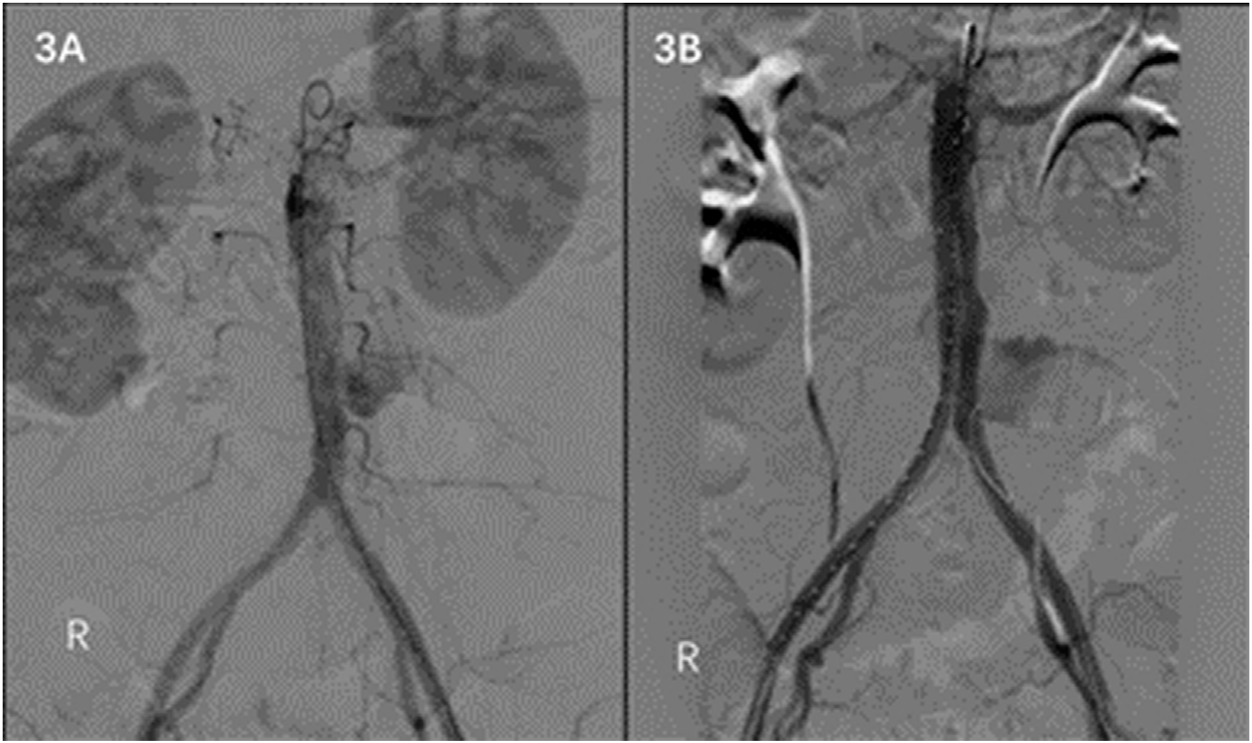
**(A)** DSA shows pseudoaneurysm formation in the middle and lower abdominal aorta. **(B)** No endoleaks on DSA after EVAR is noted.

A 58-year-old female was admitted to the emergency department on 30 July 2021 due to sudden abdominal pain for 6 h. The patient underwent abdominal enhanced CT in a local hospital, which showed abdominal aortic pseudoaneurysm formation. The patient was previously diagnosed with brucellosis 10 years ago without regular anti-brucellosis treatment. WBC count was 14.43 × 10^9^/L, the neutrophil count was 0.90 × 10^9^/L, and CRP and procalcitonin were normal. Preoperative examination and past medical history did not support abdominal aortic pseudoaneurysm caused by immune system disease or other infectious diseases. Preoperative CT showed no apparent arterial sclerosis of the patient’s aorta, considering that the patient’s abdominal aortic pseudoaneurysm was likely caused by *Brucella* infection. Endovascular exclusion of the abdominal aorta was performed under emergency general anesthesia. Intraoperative DSA showed pseudoaneurysm formation in the middle and lower segment of the abdominal aortic aneurysm (Figure 3A). EXCLUDER-covered stent (23 mm–14 mm–140 mm; W.L. Gore & Associates, Inc. Flagstaff, AZ, United States) and iliac branch stent (16 mm–12 mm–100 mm) were used to complete endovascular exclusion of the aneurysm. The proximal end was protected from the lower edge of the most inferior renal artery to many bilateral common iliac arteries. On repeat angiography, the pseudoaneurysm at the end of the abdominal aorta was not visualized, and no endoleaks were seen (Figure 3B). The patient’s postoperative abdominal pain disappeared. Regular anti-*Brucella* therapy was provided to the patient after the surgery. At the follow-up of 12 and 18 months after the operation, there was no obvious abnormality in the abdominal aorta, and the level of antibodies against *Brucella* was normal.

**FIGURE 4:**
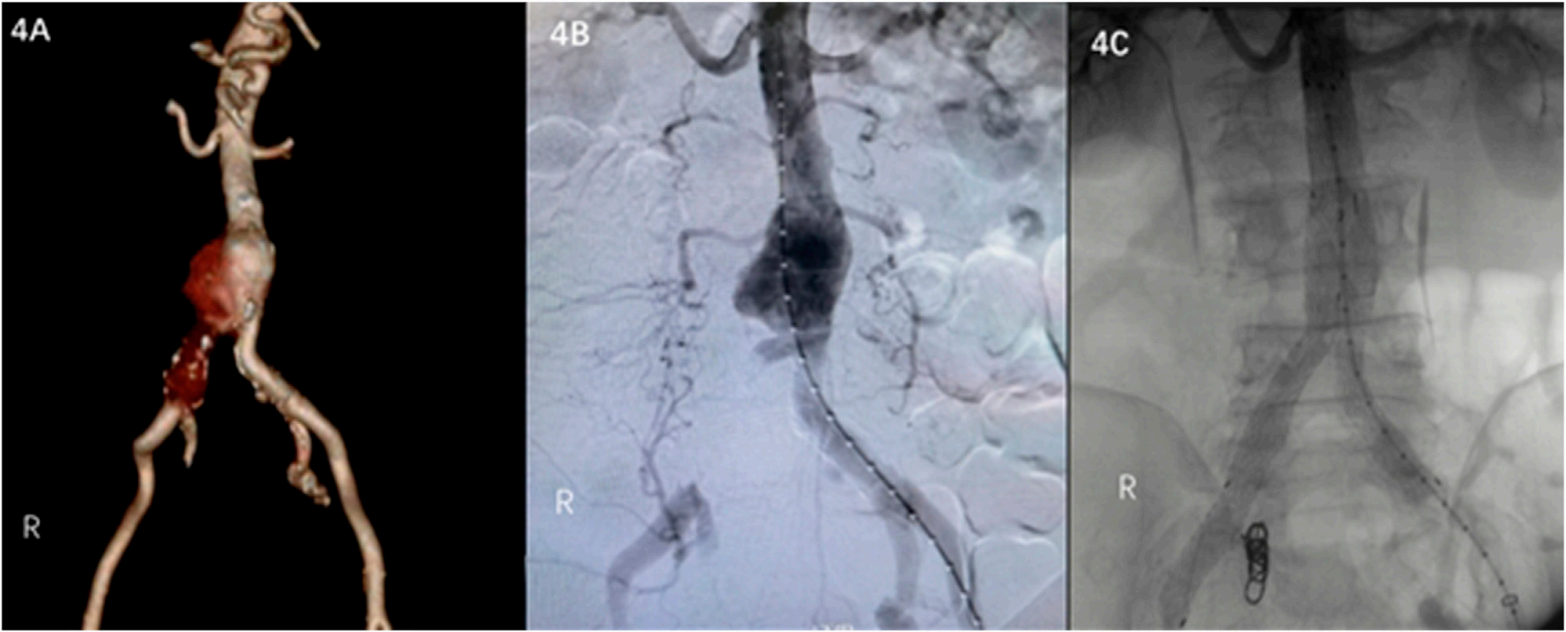
**(A)** Contrast-enhanced CT scan of the abdomen shows a pseudoaneurysm at the end of the abdominal aorta. **(B)** Intraoperative DSA shows a pseudoaneurysm at the end of the abdominal aorta and occlusion of right common iliac artery. **(C)** Intraoperative DSA shows no endoleaks.

A 65-year-old male was admitted to a hospital on 5 December 2022 due to lower abdominal and lumbar pain for half a month. Enhanced CT examination of the abdomen showed that the distal end of the abdominal aorta showed significant ballooning, which was considered an aneurysm with mural thrombus or hematoma formation (Figure 4A). The patient’s past medical history showed no history of hypertension, diabetes, coronary heart disease, surgery, trauma, drug abuse, hepatitis B, and other infectious diseases. Regarding previous work history, the patient worked and was exposed to goats for more than 5 years. The tests showed WBC of 9.89 × 10^12^/L and Hb of 121 g/L. Considering brucellosis was not excluded, a bacterial blood culture was performed. Emergency endovascular repair of the abdominal aortic aneurysm was performed, and intraoperative DSA revealed pseudoaneurysm formation at the distal end of the abdominal aorta and occlusion of right common iliac artery (Figure 4B). EXCLUDER-covered stent (23 mm–14 mm–140 mm; W.L. Gore & Associates, Inc. Flagstaff, AZ, United States) and iliac branch stent (14 mm–10 mm–100 mm) (12 mm–12 mm–100 mm) were used to complete endovascular exclusion of the aneurysm, with the proximal end starting at the inferior margin of the lowest renal artery and covering to the lots of the bilateral common iliac arteries. On repeat angiography, the terminal abdominal aortic pseudoaneurysm was not visualized, and no endoleaks were seen (Figure 4C). Three days after patient admission, *Brucella* was isolated from the patient’s blood; hence, the patient was diagnosed with brucellosis. The SAT result was 1:100, which met the solid positive result. The patient was given Doxycycline combined Rifampicin administered orally post-surgery.

## Discussion

Brucellosis is a complex disease that involves multiple systems. *Brucella* can damage the central and peripheral nervous, gastrointestinal, hepatobiliary, genitourinary, musculoskeletal, cardiovascular, and dermatological systems (Franco et al., 2007). Endocarditis is a relatively rare but potentially fatal complication of brucellosis. Although it occurs in only 1% of brucellosis patients, it is the leading cause of death in brucellosis (Pappas et al., 2005). Aortic or peripheral arterial involvement is an uncommon complication of brucellosis (Adaletli et al., 2006). Mycotic aneurysms caused by *Brucella* have been reported long ago (Aguado et al., 1987), and intravascular brucellosis affects the abdominal aorta, cerebral arteries, superior mesenteric arteries, *etc.* (Ferrero et al., 2011). *Brucella* can infect and survive in vascular endothelial cells and induce an inflammatory response associated with brucellosis vascular manifestation (Ferrero et al., 2011). Immunocompromised patients, including diabetics, alcoholics, and those on a high-fat diet or undergoing chemotherapy, are at higher risk of developing fungal aneurysms. An age of >50 years is considered a risk factor for mycotic aneurysms because of the high incidence of atherosclerotic plaque, which serves as a site of microbial attachment due to the irregular surface of the intima (Betancourt et al., 2007).

The management of mycotic aneurysms remains a challenging clinical problem for vascular surgeons, with high mortality and recurrence rates. The traditional treatment for mycotic aneurysms is resection of the aneurysm and removal of the surrounding infected inflammatory tissue, followed by *in situ* repair or extra-anatomic bypass (Sörelius et al., 2016). Muller et al. have also reported a preference for conventional procedures, supported by debridement of infected tissue and *in situ* repair with antibiotic-soaked grafts (Muller et al., 2001). However, endovascular aneurysm repair (EVAR) for mycotic aortic aneurysms simplifies the surgical procedure and provides a good alternative for this critical situation (Jones et al., 2005; Lee et al., 2006; Ting et al., 2006; Kan et al., 2007; Sörelius et al., 2014; Wanhainen et al., 2019). EVAR refers to the use of digital subtraction technology to deliver a covered stent system from the incised femoral artery and release it to the aortic lesion site, completely isolate and seal the diseased aorta and reconstruct the true lumen to achieve and restore necessary blood flow. Compared with open surgery, EVAR has the advantages of less injury, avoiding surgical bypass, interfering with respiratory function, and massive blood transfusion (Chan et al., 2005). Karl et al. investigated the survival of patients with mycotic abdominal aortic aneurysms treated by conventional surgery and EVAR across Sweden and found that although the role of EVAR for mycotic abdominal aortic aneurysms has been questioned because of the perceived risk of reinfection, EVAR for mycotic abdominal aortic aneurysms is associated with better short-term survival in comparison with conventional surgery, and comparable long-term results in terms of survival and infection related complications or reinterventions (Sörelius et al., 2016). Although *Brucella* belongs to inert bacteria, clinicians should still be cautious during preoperative, perioperative, and postoperative anti-infective treatment. Otherwise, it will lead to failure of local surgery and aggravation of systemic infection and other complications. However, the following issues need to be focused on and discussed for EVAR surgery for such disease: 1) the choice of operation time; 2) the problem of infection recurrence at the lesion site after the implantation of the covered stent; and 3) whether the stimulation of the proximal and distal ends of the stent to the autologous blood vessel induces new lesions. These four cases were all operated for the first time after the definite diagnosis of abdominal aortic pseudoaneurysm, among which one patient that was diagnosed with *Brucella* infection before the development of abdominal aortic pseudoaneurysm was systematically treated. The operation was performed during the inactive period of *Brucella* infection, and *Brucella* infection was continuously monitored and treated under the guidance of a specialist after the operation. The remaining three cases were diagnosed as brucellosis only after postoperative examination and were systematically treated under the direction of infection specialists. Because of the difficulty in assessing the extent of brucellosis damage to the native artery, we tried to extend the graft coverage to the native artery distally and proximally to the pseudoaneurysm during EVAR if possible, providing a safe landing zone (the proximal coverage length was 5cm–6 cm without affecting the renal artery blood flow; the coverage length of the distal end was 3cm–6 cm), and the oversize of abdominal aortic main body stent was between 10% and 20%. The shortest follow-up of these four cases was 1 month, and the most extended follow-up was 18 months. No local stent graft damage due to infection and no new lesions of native arteries distal and proximal to the stent were found. Before this, our center had used TEAR technology to treat one case of thoracic aortic pseudoaneurysm caused by *Brucella* infection. During the 5-year follow-up after surgery, no covered stent damage and new lesions of native arteries distal to and proximal to the stent were found.

In conclusion, under the premise of systemic treatment of *Brucella* infection, EVAR should be considered as a surgical treatment of abdominal aortic pseudoaneurysm caused by *Brucella*. During the operation, the coverage of the autologous artery at the distal and proximal ends of the pseudoaneurysm should be increased as much as possible, and the graft oversize should not be too large. Despite the good outcomes of the reported four cases, we could not decide whether EVAR is a better option than open surgery when treating *Brucella*-caused pseudoaneurysms due to the small number of cases, short follow-up, and the inability to study the long-term post-surgery results from EVAR surgery. We hope to provide long-term follow-up results from these cases and see more reports on brucellosis in the future.

## Data Availability

The original contributions presented in the study are included in the article/Supplementary Material, further inquiries can be directed to the corresponding authors.
